# Adequate diagnosis of the cause of Parkinsonism and treatment in an elderly patient with schizophrenia: A case report

**DOI:** 10.1002/pcn5.71

**Published:** 2023-01-09

**Authors:** Daisuke Yoshioka, Takehiko Yamanashi, Masaaki Iwata

**Affiliations:** ^1^ Division of Neuropsychiatry, Faculty of Medicine Tottori University Yonago Japan

**Keywords:** DAT scans, drug‐induced parkinsonism (DIP), levodopa‐responsive Parkinson's syndrome, parkinsonism, schizophrenia

## Abstract

**Background:**

Parkinsonism is frequently observed in patients with schizophrenia, and most patients are diagnosed with drug‐induced parkinsonism. However, comorbidity with idiopathic Parkinson's disease or Parkinson‐plus syndrome is also possible. The pathophysiology and treatment for each of these are entirely different, thus an appropriate diagnosis is required. However, distinguishing them based on clinical symptoms alone is often difficult, and many cases are misdiagnosed. Additionally, Parkinsonism is frequently mistaken for negative symptoms.

**Case Description:**

We report a case of 68‐year‐old woman diagnosed with schizophrenia, who was admitted to a welfare center. At approximately age 60, the patient experienced motivation reduction, a loss of appetite, and pain in the extremities. In her mid‐60s, tremor and muscle rigidity appeared; nuclear medicine testing was performed for a detailed examination, resulting in a diagnosis of levodopa‐responsive Parkinson's syndrome. Notably, the patient's parkinsonism and emotional symptoms, which had been considered negative symptoms thus far, improved with levodopa treatment.

**Conclusion:**

This case report illustrates the importance of properly diagnosing the cause of parkinsonism in patients with schizophrenia.

## Graphical abstract

A 68‐year‐old female with schizophrenia presented with parkinsonism in her mid‐60s. Based on the course of the disease and physical examination, drug‐induced parkinsonism was unlikely, and a nuclear medicine examination was performed, resulting in a diagnosis of levodopa‐responsive Parkinson's syndrome. Treatment with levodopa improved not only motor symptoms but also emotional symptoms, which had been considered negative symptoms.

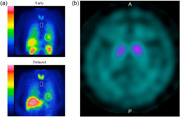

## BACKGROUND

Pharmacological treatment of schizophrenia relies primarily on the administration of antipsychotics based on the dopamine hypothesis, and during the course of this treatment parkinsonism develops frequently, occurring in ~20%–35% of patients taking antipsychotic medications.^1^, ^2^, ^3^ Additionally, after idiopathic Parkinson's disease (PD), drug‐induced parkinsonism (DIP) is the second most prevalent type of parkinsonism in older people.^4^ In most cases, therefore, parkinsonism is clinically diagnosed as DIP that emerges during the treatment of schizophrenia and is managed with reduced doses of antipsychotics and/or additional anticholinergics. Although most patients recover from parkinsonism when the administration of the offending drug is discontinued, it may take several months to completely cure it and it may persist in some patients. In cases where it persists, it is necessary to consider the possibility of other Parkinson's syndromes, including PD, corticobasal syndrome (CBS), or progressive supranuclear palsy (PSP), emerging from the offending drug, and further treatment options may be required. In addition, schizophrenia relapse is common after the dose reduction of antipsychotics; in fact, it has been reported that after each relapse one in six participants did not remit from the episode.^5^ Consequently, this knowledge makes us as practitioners hesitant in reducing the dose of antipsychotics without due consideration. Therefore, distinguishing between DIP and other parkinsonisms is crucial, although it is often challenging because of their similar symptoms.^6^, ^7^

Dopamine transporter (DAT) imaging approaches (DAT scans) have been reported to help distinguish DIP by comparing the symmetry of radiotracer uptake in the striatum.^8^, ^9^, ^10^ However, DAT scans are not fully implemented because they are not readily accessible. Furthermore, parkinsonism has clinical similarities with negative symptoms in schizophrenia, and the misdiagnosis of parkinsonism as negative symptoms can lead to an incorrect therapeutic approach with excessive dopamine blocker loading.^11^ Hence, the aforementioned perspectives necessitate a proper diagnosis and treatment for parkinsonism in patients with schizophrenia.

Herein, we report the case of an elderly female patient with schizophrenia and parkinsonism who had been using antipsychotics for many years. DAT scanning was performed, and we diagnosed her with levodopa‐responsive Parkinson's syndrome. Furthermore, the levodopa used in the treatment improved not only her parkinsonism but also her emotional symptoms.

## CASE DESCRIPTION

The female patient's first clinical presentation was at age 38, with delusions of persecution and disorganized behaviors. She was first hospitalized and diagnosed with schizophrenia after a passerby pointed out her abnormal behavior. Three years later, she was transferred to police custody and was readmitted to the hospital. She returned to her parent's home 7 months after hospitalization for recuperation and started to visit our hospital. The patient was administered haloperidol (9 mg), propericiazine (15 mg), levomepromazine (25 mg), and trihexyphenidyl hydrochloride (6 mg). She had a husband and two sons; however, the couple separated, and their children were put into an institution when she moved out. Notably, the patient has no medical or family history.

At age 60, she started showing decreased motivation, loss of appetite, and pain in her extremities and was hospitalized six times in 1 year at an internal medicine department. Subsequently, she was admitted to a psychiatric hospital for rehabilitation, and repeated falls made it difficult for her to live at home. She eventually entered a welfare center after discharge, and a neurologist from our hospital made house calls to her. In her mid‐60s, she began to experience resting tremors and muscle stiffness of the right upper limbs, and hypophonia. Additionally, she developed strong contractures of joints in the left half of her body, which forced her to use a wheelchair. A neurologist diagnosed the motor symptoms as DIP and followed them up, but they gradually worsened. Thus, she was referred to us to review the diagnosis and receive treatment. When she visited us at age 68, she was on aripiprazole (6 mg), valproic acid (200 mg), and biperiden (2 mg), and these medications had not been changed for at least 5 years. We observed negative symptoms, including blunted affect, poverty of thought, and loss of motivation. She scored 27 on the negative symptoms subscale of the Positive and Negative Syndrome Scale (PANSS). Her facial expression was stiff, but it was unclear whether this was due to negative symptoms or parkinsonism. She denied having hallucinations and delusions, and no positive symptoms were identified.

**Figure 1:**
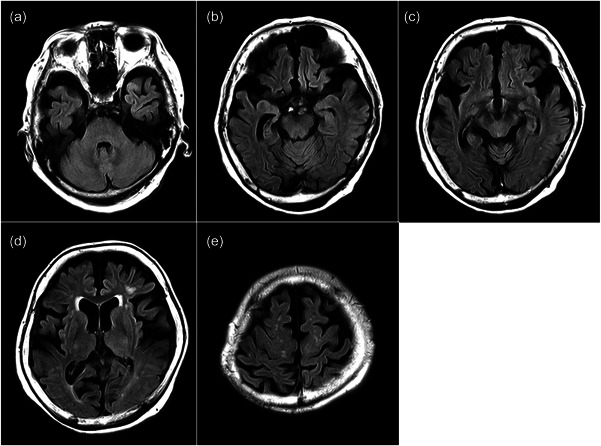
Fluid‐attenuated inversion recovery sequence brain magnetic resonance imaging showed whole‐brain atrophy, in which atrophy of the medial temporal lobe and the parietal lobe showed an asymmetric pattern; mild atrophy of the midbrain tegmentum was also observed. No vascular lesions were found in the basal ganglia. Axial brain slices at the level of (a) pons, (b) hippocampus, (c) midbrain, (d) basal ganglia and (e) central sulcus.

It was unlikely that the tremor indicated DIP because of its late onset after decades of taking an antipsychotic, and it was seen at rest, was pill‐rolling‐like, and had significant laterality. We performed magnetic resonance imaging brain scans and observed whole‐brain atrophy, in which atrophy of the medial temporal lobe and the parietal lobe showed an asymmetric pattern; mild atrophy of the midbrain tegmentum was also observed. No vascular lesions were found in the basal ganglia (Figure 1).

**Figure 2:**
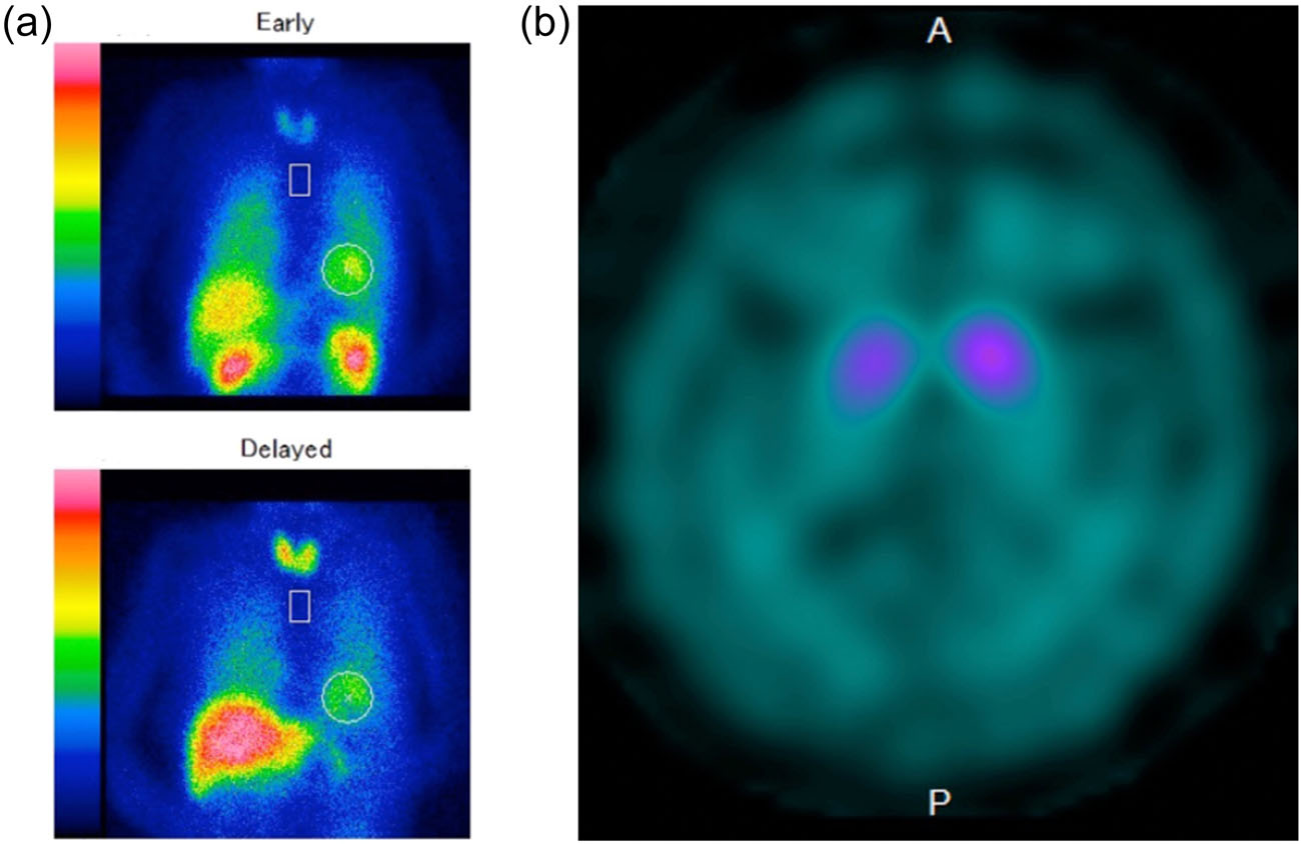
(a) 123‐I‐metaiodobenzylguanidine myocardial scintigraphy revealed no abnormalities. (b) Dopamine transporter imaging showed decreased in the bilateral putaminal specific binding ratios (2.73 and 4.28 for the right and left, respectively).

Although valproic acid was discontinued for 1 month based on the possibility of DIP, her condition did not improve. Dysphagia developed after discontinuation of biperiden for a re‐evaluation of medications, and levodopa (200 mg) was administered after consultation with the neurologist with the possibility of PD. The aripiprazole dose was maintained at the same level for some time because of concerns regarding possible exacerbation of psychiatric symptoms. The patient's tremor and dysphagia improved when the levodopa dose was increased to 300 mg. In addition, her motivation, mood, and facial expression improved, and she began to joke. Scoring using the PANSS revealed a decreased negative symptom score of 17. 123‐I‐metaiodobenzylguanidine (MIBG) myocardial scintigraphy and DAT scans were performed to differentiate the type of parkinsonism. MIBG myocardial scintigraphy revealed no abnormalities (Figure 2a), whereas DAT scans showed decreased bilateral putaminal specific binding ratios (2.73 and 4.28 for the right and left, respectively) (Figure 2b). PD was listed as a differential diagnosis because of the presence of pill‐rolling tremor and levodopa‐responsive parkinsonism. CBS and PSP were also listed as a differential diagnosis because of a history of repeated falls asymmetric clinical findings and brain atrophy, atrophy of the tegmentum midbrain, and nuclear medicine findings. Although a definitive diagnosis could not be determined, we considered her pathology to be levodopa‐responsive Parkinson's syndrome. Strong contractures were assumed to result from dystonia.

Six months after starting levodopa, an increased online shopping frequency was observed, and a manic episode was suspected. However, we did not consider it a side effect of levodopa and kept her under observation because it was transient. Since then, her motor function and psychiatric symptoms have remained stable, and she has not experienced any side effects of levodopa.

## DISCUSSION

Antipsychotic medications are often used to treat schizophrenia and bipolar disorder, and their use has recently expanded to include patients with dementia.^12^ A recent epidemiologic study on parkinsonism showed that typical antipsychotics, but not atypical antipsychotics, were identified as the cause of most drug‐induced cases.^13^ However, another retrospective study on the older population showed that 46% of parkinsonism was due to atypical antipsychotics.^7^ Although the study findings are inconsistent, DIP remains a concern for antipsychotic treatment.

Nevertheless, there are various pathologies that cause parkinsonism, including idiopathic PD, Parkinson‐plus syndrome, and secondary parkinsonism. The Parkinson‐plus syndrome comprises a group of disorders, including CBS, PSP, dementia with Lewy bodies, and a variety of less common disorders. Secondary parkinsonism is caused by drugs, vascular diseases, infections, metabolic abnormalities, and toxins. The most common drug‐induced causes are antipsychotics, but other medications include antiemetics, antiepileptic drugs (e.g., valproic acid), and many others.^8^ It is necessary to differentiate between these parkinsonism causes and take appropriate action.

DIP often develops in patients treated with antipsychotics, with latency ranging from a few days to several years after medication exposure,^14^ although most patients recover from DIP within a few months after drug withdrawal.^15^ Clinically, DIP is typically characterized by the absence of tremors and bilateral and symmetric parkinsonism, with more prominent bradykinesia and rigidity than in patients with PD.^4^, ^16^, ^17^ However, it has been reported that patients with DIP demonstrate similar phenotypes to PD (i.e., asymmetric rest tremor)^18^ and numerous studies have suggested that clinical indicators alone cannot be used to differentiate DIP from other Parkinson's syndromes.^8^ A study that investigated patients with schizophrenia who developed parkinsonism revealed that only those with abnormal DAT scans showed worsening motor symptoms, and only that group responded to levodopa treatment.^19^ This result suggests that a degenerative disease might underlie parkinsonism in some patients with schizophrenia who are chronically exposed to antipsychotics. However, it is difficult to distinguish the pathophysiology from clinical findings alone. Therefore, functional imaging of the DAT can be helpful for identification. In this case, we considered the possibility of DIP to be low because the patient had a noticeable bilateral difference and, more importantly, owing to the late onset of parkinsonism after several decades of taking antipsychotics. We performed nuclear medicine testing, enabling us to diagnose parkinsonism as levodopa‐responsive Parkinson's syndrome without the risk of schizophrenia recurrence after discontinuing the antipsychotic medication. In our case, parkinsonism was improved by treatment with levodopa, which was used appropriately given the diagnosis. However, it remains possible that antipsychotic drugs may cause some loss of neurites, synaptic spines, or synapses in the cortical structures, especially when used at high dose for long periods of time.^20^ In fact, a recent positron emission tomography study targeting vesicular monoamine transporter type 2 (VMAT2) binding sites reported that striatal VMAT2 binding was abnormal in a subset of chronic DIP cases and differs in spatial distribution from PD.^21^ These reports may influence the concept of parkinsonism caused by antipsychotic drugs in the future.

Notably, the patient's mood and motivation improved with levodopa therapy; nevertheless, it was difficult to distinguish whether the improvement in her emotional symptoms was due to a reduction in parkinsonism or an improvement in negative symptoms. A recent systematic review and meta‐analysis did not show the efficacy of levodopa on negative symptoms.^22^ However, her facial expressions improved, she began to joke, and she even started shopping despite her autistic lifestyle. Based on these facts, we believe that levodopa contributed, at least partially, to improving her negative symptoms. In addition, although the safety of levodopa use in patients with schizophrenia is always a matter of caution, a meta‐analysis reported that psychiatric symptoms worsened in <20% of patients.^23^ Even in patients with schizophrenia, the use of levodopa should be considered in appropriate cases.

Summarily, parkinsonism is often encountered in treating schizophrenia, although it has a wide variety of causes. Proper diagnosis and treatment of the underlying cause using nuclear medicine testing and evaluating symptoms and clinical course can prevent inappropriate interruption of schizophrenia treatment and the appearance of adverse events, which is very beneficial to the patient's prognosis.

## AUTHOR CONTRIBUTIONS

Daisuke Yoshioka treated the patient and drafted the manuscript. Takehiko Yamanashi and Masaaki Iwata critically reviewed the draft and revised it. All authors approved the final version of the manuscript.

## CONFLICT OF INTEREST

The authors declare no conflict of interest.

## ETHICS APPROVAL STATEMENT

This study was conducted according to the principles of the Declaration of Helsinki.

## PATIENT CONSENT STATEMENT

Informed written consent and a signed release were obtained from the patient for publication of this report and any accompanying images.

## CLINICAL TRIAL REGISTRATION

Not applicable as this is a case report.

## Data Availability

Data sharing is not applicable to this article.
